# Effects of Horticultural Activities on Attitudes toward Aging, Sense of Hope and Hand–Eye Coordination in Older Adults in Residential Care Facilities

**DOI:** 10.3390/ijerph18126555

**Published:** 2021-06-18

**Authors:** Hui-Ying Chu, Hui-Shan Chan, Mei-Fang Chen

**Affiliations:** 1 Department of Living Services Industry, Tainan University of Technology, No. 529, Zhongzheng Rd., Yongkang District, Tainan City 71002, Taiwan; 2 Department of Applied Cosmetology, National Tainan Junior College of Nursing, 78 Sec. 2, Minzu Rd., Tainan City 70043, Taiwan; shan033@mail.ntin.edu.tw; 3 Department of Nursing, National Tainan Junior College of Nursing, 78 Sec. 2, Minzu Rd., Tainan City 70043, Taiwan; meifang0302@gmail.com

**Keywords:** horticultural activity, attitudes toward aging, sense of hope, hand–eye coordination

## Abstract

This study investigated the effects of an 8-week horticultural activity intervention on attitudes toward aging, sense of hope, and hand–eye coordination in 88 older adults in residential care facilities. In the experimental group, the mean score for “attitudes toward aging” increased from 3.81 before the intervention to 4.74 points after the intervention (standard deviation SD = 0.24 and 0.27, respectively), and the control group dropped from 3.75 to 3.70 (standard deviations, respectively SD = 0.27 and 0.28). The mean score for “sense of hope” increased from 3.28 before the intervention to 3.81 points after the intervention (SD = 0.49 and 0.26, respectively). In contrast to the control group, the mean score gradually declined from 3.26 to 3.16 points (standard deviation SD = 0.54 and 0.48, respectively). In the test of hand–eye coordination, the time required to complete the cup stacking test significantly decreased from 33.56 to 25.38 s in the experimental group but did not significantly change in the control group. Generalized estimating equation analysis revealed a significant interaction between group and time (*p* < 0.001). The data trends revealed significant differences in outcomes between the experimental group and the control group. At 3 months after the end of the study, the effect size in the experimental group remained higher than that in the control group.

## 1. Introduction

Advances in medical and health technology have increased mean life expectancy and have made population aging a topic of global relevance. As of April 2019, Taiwan had officially transitioned to an aged society, with older adults accounting for 14.81% of the total population [1]. This proportion is expected to increase to 36.6% by 2050; that is, 4 out of 10 people will be aged 65 years and older by 2050 [2]. Countries facing population aging must endeavor to maintain the physical and mental health of older adults and slow down the aging process to relieve the financial burden of medical resource consumption by this age group.

In older adults, attitudes toward aging, i.e., attitudes resulting from their subjective experience of the aging process, comprise their attitudes regarding aging cognition, aging emotions, and aging behavior [3]. McConatha et al. (2004) asserted that individuals experience fear and anxiety when they become aware that they are aging [4]. Fear and anxiety result from their increasingly negative perceptions of their attractiveness, competence, etc. These negative attitudes can then impair their physical health by weakening their immunity and resistance to disease [5,6,7,8,9]. Other adverse impacts of these attitudes can include decreases in mobility, life satisfaction, and happiness [10,11,12,13,14]. Therefore, researchers have used attitudes toward aging as a psychological variable for predicting death in older adults [8,15]. In contrast, individuals who perceive aging as a natural and inevitable process tend to accept the reality of aging rather than attempt to alter or control it. Older adults with positive attitudes toward old age not only tend to have good health and quality of life, but they also tend to have a long life expectancy [16,17,18]. Thus, a positive attitude toward aging plays a pivotal role in quality of life for older adults. 

A sense of hope, which is a subjective state of mind and a representation of inner strength, gives individuals positive expectations of attaining things they desire in life or achieving important goals. People with hope are convinced that a bright future awaits them [19]. A strong sense of hope also gives people the strength and courage to face and overcome difficulties [20]. In contrast, a sense of despair causes negativity and loss of the courage needed to thrive or even survive. Older adults experience various stresses (e.g., physical diseases, impaired motor function, and psychological problems) as they age [21,22], which often cause them to lose their vitality, their hope, and their positive expectations about the future. Individuals who experience these negative changes may eventually develop depression and lose their sense of purpose in life [23,24]. Such negative emotional states are particularly common in older adults living in residential care facilities [25,26]. Studies of older adults indicate that hopelessness is a strong mortality predictor, even when controlling for age-associated increases in risks of depression and other medical conditions in older age groups [27,28,29,30]. Therefore, a sense of hope is essential for wellbeing in older adults.

Hand–eye coordination refers to the control of eye and hand movement and the process in which they work in concert. Information from environmental stimuli that is transmitted to the nervous system is processed in the cerebral cortex, which then triggers hand muscles to perform the most appropriate movement at the most appropriate time [31,32,33]. Older adults require adequate hand–eye coordination to perform activities of daily living such as eating and getting dressed. Since hand–eye coordination is a fundamental aspect of physical capacity in older adults [34], delaying age-related loss of hand–eye coordination should be considered a key objective of any healthcare system.

Horticultural therapy is a mild, noninvasive adjuvant therapy suitable for a broad spectrum of individuals with widely varying characteristics (e.g., varying age, disability, etc.). Studies of horticultural therapy reveal that simply viewing flowers and plants (e.g., fresh pansies) can decrease sympathetic nerve activity and induce feelings of comfort, relaxation, and happiness [35]. Horticultural activities can also reduce salivary cortisol levels and relieve acute stress [36]. In a work environment, substantial greenery can reduce psychological stress [37]. 

Moreover, horticultural activities can confer neuroprotective effects. A recent study reported that an experimental group of elderly people who participated in horticultural activities had significantly higher brain-derived neurotrophic factor levels compared to a control group of elderly people who did not receive the horticulture intervention. High levels of brain-derived neurotrophic factor and platelet-derived growth factor [38,39] result in high levels of anti-inflammatory factors in the blood and enhanced neuroprotective function [40]. Thus, the therapeutic psychological and physiological effects of horticultural activities are empirically well established. According to the literature, attitudes toward aging, sense of hope, and hand–eye coordination are directly or at least indirectly related to the above physiological and psychological benefits. Therefore, the current study explored the influence of horticultural activities on attitudes toward aging, sense of hope, and hand–eye coordination. 

In addition to the above neuroprotective/psychological benefits, many horticultural activities (e.g., watering plants, pulling weeds, and picking up fallen leaves) have substantial physical health benefits because they require physical movement that exercises motor skills. Specifically, such activities can benefit movement symmetry, muscle strength, vestibular function, and hand–eye coordination in older adults and individuals with disabilities. Well-documented psychological benefits of horticultural activities in older adults include improved concentration and decreased negative emotions (e.g., fear of poverty) [41,42,43,44,45,46,47,48,49,50,51,52,53,54]. The benefits of these activities also extend to enhanced personal life satisfaction and mental health. 

Since the practice of horticultural therapy in Taiwan is still at an early stage, most studies performed in older adults in Taiwan have focused on those who are healthy and still living in the community. For convenience and safety, older adults who are physically weak or living in residential care facilities are often excluded from participation in research [55] despite their potential for having complex mental issues and their urgent need for attention and care. Moreover, the small sample sizes and short follow-up periods in most studies performed in Taiwan limit the ability to draw conclusions regarding the optimal duration of interventions or conclusions about their therapeutic efficacy. Based on the literature review, this research proposes the hypothesis that horticulture activities have a positive effect on sense of hope and hand–eye coordination ability of the elderly. Furthermore, the study developed a horticultural intervention for older adults in residential care facilities and evaluated its effectiveness at 3 months post-intervention. The findings may be applicable in guiding the use of green resources as an effective approach to promoting mental and physical health in older adults in residential care facilities. 

## 2. Materials and Methods

### 2.1. Design

This study was approved by the Human Experiment and Ethics Committee of National Cheng Kung University (approval number: 108-417-2). The experiment was performed from December 2019 to August 2020. A nonequivalent pretest–posttest control group design was used in this quasi-experimental study. Questionnaire surveys were used for data collection. The participants, 90 older adults from 3 residential care facilities, were randomly assigned to experimental and control groups. A pretest was administered before the intervention. Over an 8-week period, the experimental group attended eight 2 h sessions of horticultural activities, whereas the control group engaged in (their typical daily) leisure activities (e.g., watching television, listening to music, and reading newspapers). After the intervention, a posttest was administered to compare the two groups in attitudes toward aging, sense of hope, and hand–eye coordination. Finally, a follow-up test was administered 3 months after the intervention to determine the duration of its therapeutic effect.

### 2.2. Recruitment

Older people were recruited by convenience sampling from three nursing homes in Tainan, Taiwan. The authors first performed a 3-month pre-intervention study to confirm its feasibility. An announcement was then posted on bulletin boards at the three residential care facilities to provide details of the study and to invite the residents to participate. The inclusion criteria were as follows: (1) age 65 years or older, (2) ability to communicate and express feelings clearly, (3) clear consciousness, full mobility, and self-care ability, and (4) no diagnosis of a psychiatric condition. Social workers in residential care facilities used the Mini-Mental State Examination to screen potential participants and then recommended eligible residents to the researchers. Residents diagnosed with cognitive impairment in the examination (score ≤ 24) were excluded because they were expected to lack the ability to participate in horticultural activities, which would have caused an interference effect. The G*Power analysis program was used to estimate the required sample size. The means and standard deviations (SDs) in 30 pilot test samples yielded an effect size of 0.86. Based on the Cohen (1992) equations α = 0.05 and 1 − β = 0.95, the minimum sample size was 74 (i.e., 37 participants per group) [56]. To compensate for potential attrition, a sample larger than the minimum (90 participants) was recruited. The participants were alternately assigned to the experimental and control groups (each of which comprised 15 residents in the same care facility). A total of 90 participants agreed to participate in the study. After the pretest, a research assistant who had not participated in data collection or data analysis assigned the participants to experimental and control groups. To randomize the group assignments, the research assistant wrote the room number of each participant on a slip of paper and placed all slips in a test tube. The slips were then randomly drawn from the test tube one at a time, and each patient was alternately assigned to the experimental group or control group. 

**Figure 1:**
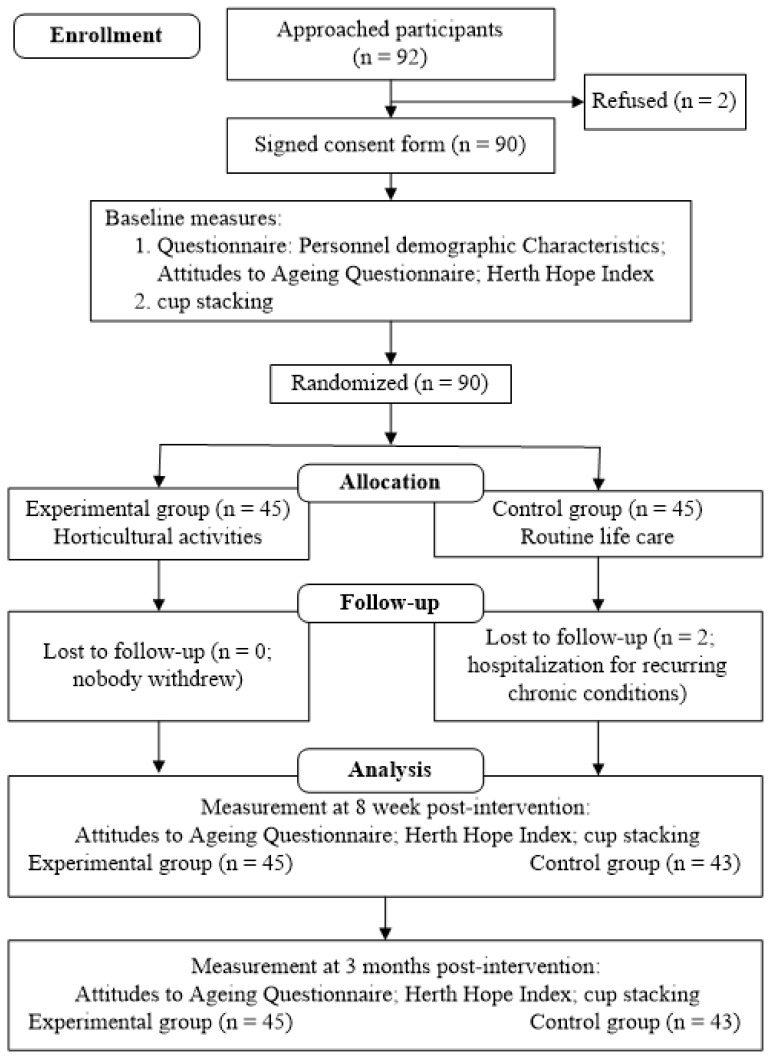
Flowchart of procedures for recruiting participants, implementing the intervention, and administering the Attitudes to Ageing Questionnaire, Herth Hope Index, and cup stacking task.

Each group originally contained 45 participants; however, 2 participants in the control group withdrew due to chronic diseases. Therefore, the total number of participants was 88. The retention rates of the experimental and control groups at T2 (follow-up test) were 100% and 95.6%, respectively. Figure 1 presents the procedure for participant recruitment, pretesting, activity intervention, and measurement.

### 2.3. Intervention

Multiple publications (e.g., [57,58,59]) were referenced during preparation of lesson plans for the intervention, which was entitled “Everlasting Greenery: Horticultural Experience.” Each intervention session comprised three activities: a warm-up activity, a horticultural activity, and a group sharing activity. To exclude the potential impact of severe weather events on the intervention, all activities were performed indoors. The principal researcher and four research assistants explained the procedures for the activities to the participants. The principal researcher then assigned three participants in the experimental group to work with each researcher. The activity goals were as follows: (1) to revitalize the nervous system by stimulating the senses of touch, smell, and sight (sessions 2, 4, 5, 6, 7, 8); (2) to improve hand–eye coordination (all sessions); (3) to induce positive attitudes toward aging and a sense of accomplishment by completing the horticultural activity (all sessions); (4) to induce a sense of hope through observation of plant growth (sessions 1, 3, 4, 7); and (5) to strengthen friendships among residents by encouraging interpersonal interactions (all sessions). For participants who were absent from activities for personal reasons, the research team contacted care facility managers and social workers to determine the reason for the absence. At the end of each session, the research team worked with participants who had been absent to ensure that they could continue to participate in the activity with their peers. Table 1 presents the detailed content of the horticultural activity program.

### 2.4. Measures

The survey instruments used in this study were the standard questionnaire for demographic characteristics, Attitudes to Ageing Questionnaire (AAQ) [60], and the Herth Hope Index (HHI) [19]. The cup stacking task in the pretest was designed by the World Sport Stacking Association (2018) [61]. Additional details of the survey instruments are given below. 

#### 2.4.1. Standard Questionnaire for Demographic Characteristics

This questionnaire was used to collect demographic data, including age, education level, religion, marital status, and medical history.

#### 2.4.2. AAQ

The AAQ is a 24-item self-reported scale for surveying attitudes about aging. Each item is scored on a 5-point Likert scale from 1 (strongly disagree) to 5 (strongly agree). The minimum and maximum scores are 24 and 120 points, respectively. Confirmatory factor analysis was performed to determine scale validity; the comparative fit index was 0.842, χ^2^ was 4559.9, df was 248, and root mean square error of approximation was 0.056. The three dimensions of the AAQ are (1) psychosocial loss, defined as the psychological and social losses and negative aspects of aging experienced by older adults (α = 0.807); (2) physical change, defined as the experience of changes related to physical function, health, exercise, and aging (α = 0.809); and (3) psychological growth, defined as the ability to perceive the benefits of aging, e.g., wisdom or progression of interacting with others (α = 0.738) [60]. Attitudes about aging are measured in three dimensions: physical change, psychological growth, and psychosocial loss. Items for the psychosocial loss dimension of the AAQ are negatively worded items and were reverse scored for statistical analysis in this study. The final score is the sum of the three subscale scores; a high score indicates a positive attitude toward aging.

The reliability of the AAQ was assessed by calculating the Cronbach’s α value. The validity of the AAQ was assessed by an expert panel comprising two nursing staff members, two social workers, and two nursing school professors who specialized in aging physiology. The content validity index was 0.85. The Cronbach’s α values for physical change, psychological growth, and psychosocial loss were 0.919, 0.952, and 0.971, respectively, indicating high reliability. The item analysis yielded *t* values ranging from 2.52 to 10.69 (*p* < 0.05), indicating satisfactory discriminatory power. 

#### 2.4.3. HHI

Sense of hope was evaluated by HHI, an abbreviated version of the Herth Hope Scale. The 12 items on the HHI are scored on a 4-point Likert scale from 1 (strongly disagree) to 4 (strongly agree). Items 3 and 6 are reverse scored. The minimum and maximum scores are 12 and 48 points, respectively, and a high score indicates a strong sense of hope. The three dimensions of the HHI are (1) inner sense of temporality and future, which is defined as the perception that a positive, desired outcome is realistically probable in the near or distant future (items 1, 2, 6, and 11); (2) inner positive readiness and expectancy, which is defined as a feeling of confidence in initiating plans to affect the desired outcome (items 4, 7, 10, and 12); and (3) interconnectedness with self and others, which is defined as recognition of the interdependence and interconnectedness between self and others and between self and spirit (items 3, 5, 8, and 9). The Cronbach α value of the original HHI was 0.97, and the retest reliability was 0.91. In terms of criterion-related validity, its correlation coefficient (r) with multiple similar scales was 0.92–0.81, and its correlation coefficient (r) with the Hopelessness Scale was −0.73, indicating satisfactory construct validity [62]. In the current study, the HHI had a Cronbach’s α value of 0.88. Expert validation of the HHI indicated a content validity index of 0.85. Item analysis yielded *t* values ranging from 3.14 to 15.00 (*p* < 0.05), indicating satisfactory discriminatory power. 

#### 2.4.4. Cup Stacking Test

**Figure 2:**
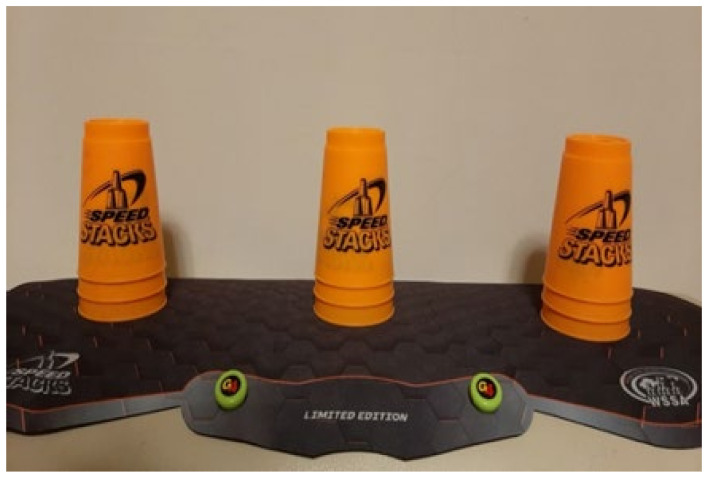
Start with three stacks of three cups.

**Figure 3:**
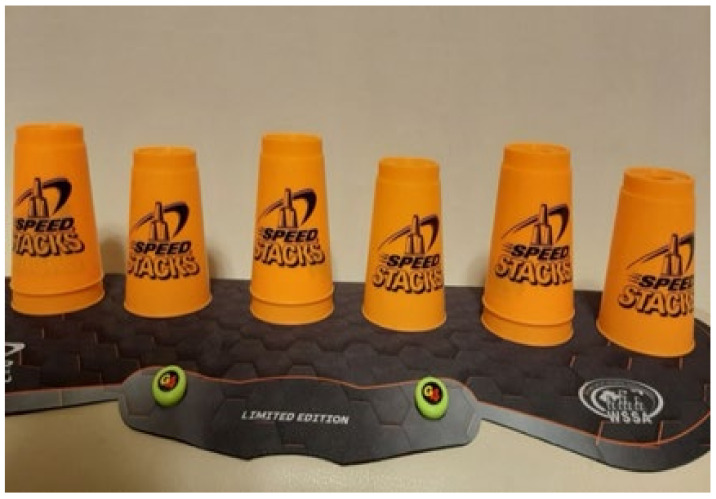
From left to right, lift the cup at the top of each stack with the right hand, and set it next to the cup at the bottom of the stack.

**Figure 4:**
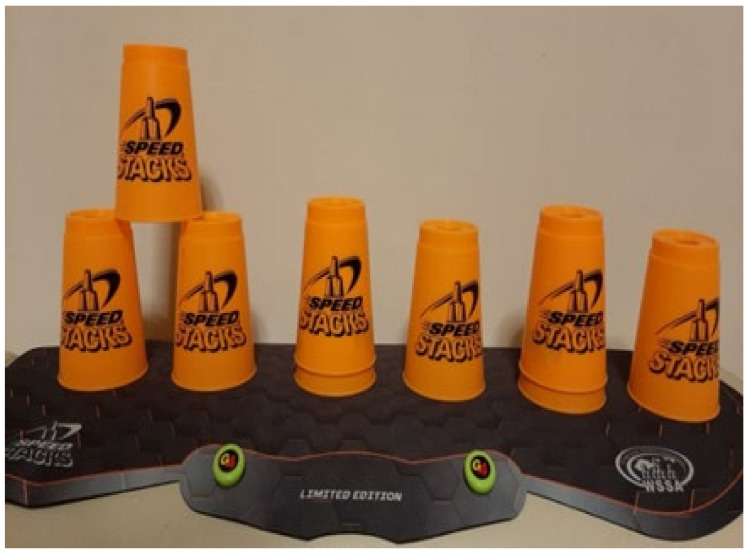
Lift the cup at the top of the stack of two cups on the left and place it on top of the two bottom cups to form a pyramid.

**Figure 5:**
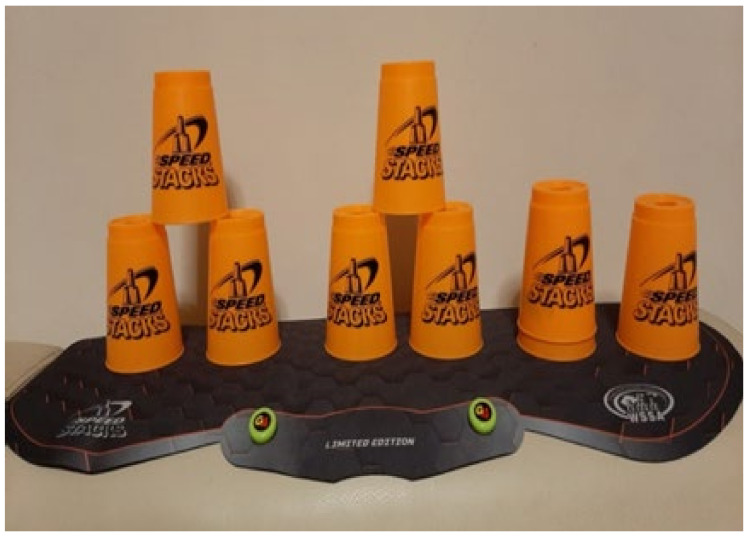
Form a pyramid from the cups in the middle.

**Figure 6:**
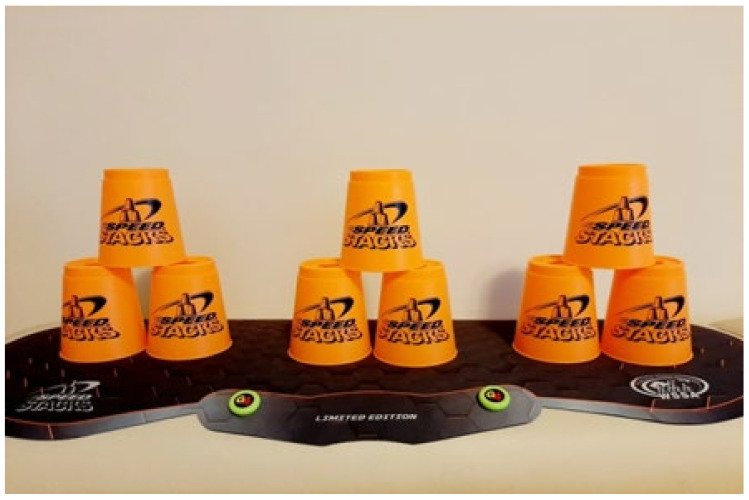
Form a pyramid from the cups on the right.

**Figure 7:**
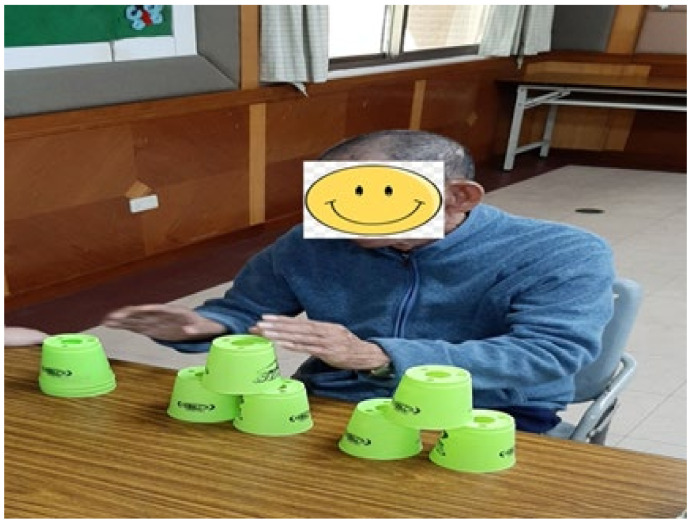
A participant stacking cups.

The cup stacking task was administered as follows. Divide nine cups into three stacks with a 15 cm distance between stacks. Place the stacks on a table 30 cm in front of the participant (Figure 2). Read out loud the instructions for the following stacking tasks (Figure 2, Figure 3, Figure 4, Figure 5, Figure 6 and Figure 7). Figure 7 shows a participant stacking the cups. Faster completion of the task indicates better hand–eye coordination.

Expert validation (by six experts) was performed to assess the validity and relevance of the cup stacking task. The content validity index was 0.83. The pretest data were subjected to item analysis. Satisfactory discriminatory power was indicated by a *t* value of 6.68 (*p* < 0.000). Satisfactory reliability was indicated by a Pearson *r* of 0.85 in the test–retest method. 

### 2.5. Statistical Analyses

Data were analyzed with SPSS Version 23 (IBM Corp., Armonk, NY, USA). The chi-squared test was used to examine group differences in personal characteristics. Additionally, Cohen’s *d* was used to analyze the effect size in the control and experimental groups for attitudes toward aging, sense of hope, and hand–eye coordination. For these three variables, Pearson product–moment correlation was used to analyze the correlation between the control and experimental groups. The generalized estimating equation (GEE) with a first-order autoregressive error structure (GEE AR (1)) was used to address data dependency issues. In both groups, mean scores for attitudes toward aging, sense of hope, and hand–eye coordination were compared among the pretest, posttest, and follow-up test.

## 3. Results

### 3.1. Baseline Data

Table 2 compares baseline characteristics between the experimental and control groups. Demographic and psychosocial characteristics did not significantly differ between the two groups (*p* > 0.05).

### 3.2. Analytical Results for AAQ, HHI and Hand–Eye Coordination Test

In the experimental group, the mean single-item AAQ scores for the pretest, first posttest, and follow-up test were 3.81 ± 0.24, 4.74 ± 0.27, and 4.31 ± 0.25 points, respectively. The corresponding scores in the control group were 3.75 ± 0.27, 3.70 ± 0.28, and 3.48 ± 0.34 points. Comparison of item scores among the three administrations of the AAQ revealed that, in both groups, the item with the highest score was, “I want to give a good example to younger people.” The mean scores for this item ranged from 4.98 to 4.70 (SD = 0.15 and 0.51, respectively). The item with the second highest score was, “It is very important to pass on the benefits of my experiences to younger people.” The mean scores for this item ranged from 4.93 to 4.47 (SD = 0.25 and 0.63, respectively). These results indicated that participants were very concerned about how they were perceived by the younger generation. Notably, transmission of experience is an essential element of a positive attitude toward aging. 

Trend analysis of scores for the three dimensions of the scale further revealed that, in both groups, the item with the lowest score was, “It is more difficult to talk about my feelings as I get older” in the “psychosocial loss” dimension. Mean scores for this item ranged from 2.16 to 4.62 (SD = 0.60 and 0.53, respectively). In both groups, the item with the second lowest score was, “Old age is a time of loneliness” in the “psychosocial loss” dimension. Mean scores for this item ranged from 2.24 to 4.47 (SD = 0.71 and 0.73, respectively). In older people, depression and loneliness have a strong negative impact on attitudes about aging. In the experimental group, however, the score for each item in the “psychosocial loss” dimension before the intervention was at least 1.5-2 times higher after the intervention. Compared to the control group, the experimental group also had considerably lower loneliness, depression, and perceived loss of dependence but had considerably higher sense of social participation. These changes revealed that the horticultural activities were highly beneficial to positive attitudes toward aging.

Regarding sense of hope, the experimental group had mean single-item scores of 3.28 ± 0.49, 3.81 ± 0.26, and 3.39 ± 0.40 points in the pretest, first posttest, and follow-up test, respectively. The corresponding scores in the control group were lower: 3.26 ± 0.54, 3.15 ± 0.49, and 2.97 ± 0.41 points, respectively. In both groups, the sense of hope dimension with the lowest score was the inner sense of temporality and future dimension, indicating a lack of confidence in the planning and development of their current and future lives. Comparison of item scores among the three administrations of the HHI revealed that, in both groups, the item with the highest score was, “I am able to give and receive caring and love.” Mean scores for this item ranged from 3.49 to 3.98 points (SD = 0.63 and 0.15, respectively). Notably, in the experimental group, the item with the largest increase in mean score was, “I have a positive outlook toward life.” The mean score increased from 3.24 before the intervention to 3.89 points after the intervention (SD = 0.83 and 0.38, respectively). These score changes demonstrate that the intervention effectively increased enthusiasm for life in the participants. In both groups, the item with the lowest mean score was, “I feel all alone.” Mean scores for this item ranged from 2.33 and 3.20 points (SD = 0.90 and 0.79, respectively), which was consistent with the AAQ results, i.e., most participants had feelings of loneliness, helplessness, and fear of what lay ahead. The feelings also negatively affected their sense of hope for the future.

In the experimental group, hand–eye coordination scores were 33.56 ± 15.51, 25.38 ± 12.38, and 30.79 ± 11.50 points in the pretest, posttest, and follow-up test, respectively. The corresponding scores in the control group were 36.45 ± 15.61, 37.15 ± 15.47, and 41.72 ± 14.97 points, respectively. The scores for the experimental group revealed significantly improved cup stacking speed after the intervention, which indicated improved hand–eye coordination. In the control group, however, the scores indicated that cup stacking speed did not significantly change and actually slightly declined over the course of the study. The follow-up test revealed a slight decline in reaction time in the experimental group. Table 3 presents the single-item scores for the pretest, posttest, and follow-up test. 

### 3.3. Effect Size of Horticultural Activities on Attitudes toward Aging, Sense of Hope and Hand–Eye Coordination

The means and standard deviations of the two posttest measurements of the experimental and control groups were used with Cohen’s *d* [56] to calculate effect sizes. Small, medium, and large effect sizes are defined as Cohen’s *d* values of <0.2, 0.5, and 0.8, respectively. Table 4 shows that, after the 8th week of horticultural activities and 3 months after completion of the activities, the two posttests revealed a large effect size of 0.8 in attitudes toward aging and sense of hope. For hand–eye coordination, Cohen’s *d* values for attitudes toward aging and sense of hope were −0.84 and −0.82, respectively, which indicated improved hand–eye coordination in the experimental group. The effect size for the follow-up test decreased but remained large. The scores for these three variables differed significantly between the experimental group and the control group, and the effect size in the experimental group was large (Table 4).

### 3.4. Correlation Analysis of Attitudes toward Aging, Sense of Hope and Hand–Eye Coordination

In the control group, Pearson product–moment correlation was calculated to investigate correlations among attitudes toward aging and their three dimensions in AAQ; sense of hope and its three dimensions in HHI; and hand–eye coordination. The three tests performed in the control group revealed significant (*p* < 0.01) positive associations among physical change, psychological growth, and attitudes toward aging, which indicated that attitudes toward aging tended to be positive when scores for physical change and psychological growth were high. In the control group, psychosocial loss had significant negative correlations with physical changes and social psychological growth (*p* < 0.05).

Additionally, physical change had a significant (*p* < 0.01) positive association with psychological growth, which indicated that participants who were able to accept that physical change had the high adaptability needed for psychological growth. Moreover, positive attitudes toward aging had a significant (*p* < 0.05) negative association with hand–eye coordination in the older adults in this study, which indicated that the participants maintained a positive attitude about aging despite their poor hand–eye coordination. These abilities differ in that the first is psychological and the latter is physical. For sense of hope, the correlations among its three dimensions remained high. However, the correlations among the three dimensions declined over time, particularly those for the inner sense of temporality and future dimension (correlations for the other dimensions changed from high to moderate). This implied that, compared with the other two dimensions, the prospects and plans for the future life dimension were more likely to change over time (Table 5).

In the experimental group, overall attitudes toward aging had strong positive correlations with the three dimensions (*p* < 0.01) in the pretest and first posttest. In the first posttest, the correlation between psychosocial loss and attitudes toward aging increased from moderate (*p* < 0.05) to strong (*p* < 0.01). This implies that the horticultural activities increased the correlation between sense of psychosocial loss and attitudes toward aging. However, the correlation between physical change and attitudes toward aging changed from a significant (*p* < 0.01) correlation to a nonsignificant correlation in the follow-up test. A possible explanation is that experiencing the physical effects of aging for 3 months altered the perception of physical change, which in turn affected the positive correlation between physical change and attitudes toward aging. Regarding sense of hope, the correlation between sense of hope and its three dimensions and the correlation between the subscales remained high in the three tests, particularly in the interconnectedness with self and others dimension. After the intervention, the interconnectedness with the self and others dimension had a stronger correlation with sense of hope. A possible explanation is that the interpersonal interaction during the activities reduced the sense of loneliness in the participants (Table 5).

### 3.5. Changes in Attitudes toward Aging, Sense of Hope and Hand–Eye Coordination after the Horticultural Activity Intervention

Because repeated measurement data were used, the results of a standard error analysis using a generalized estimating equation (GEE) are more accurate than those provided by a generalized linear model. The following methods were used to determine the effectiveness of the intervention. 

#### 3.5.1. Attitudes toward Aging

The main effect of the group on attitudes toward aging was nonsignificant (*p* = 0.208) whereas that of time (first posttest and follow-up test) was significant (*p* = 0.000; *p* = 0.047, respectively). Because of interactions between group and time (*p* < 0.001), pretest results for the control group were used as reference values for comparing how the groups differed in the interaction with time. In the experimental group, the mean score increased from 3.81 in the pretest to 4.74 points in the first posttest, then further decreased to 4.31. In the control group, the mean score decreased from 3.75 to 3.70 points, then further decreased to 3.48. GEE analysis revealed that, compared to the control group, the experimental group had significantly higher scores in the first posttest (*B* = 0.97, *p* < 0.001) and in the follow-up test (*B* = 0.77, *p* < 0.001). Table 6 shows that, in the experimental group, the mean score for attitudes toward aging had significantly increased—by 0.92 points (SD = 0.04)—by the end of the intervention. However, the effect of the intervention diminished over time; the corresponding score in the follow-up test was 0.43 points lower (SD = 0.05) than that in the first posttest. In the control group, the mean score for attitudes toward aging gradually decreased by 0.27 points from the pretest to the follow-up test. Table 6 shows the GEE results.

#### 3.5.2. Sense of Hope

The main effect of the group on sense of hope was nonsignificant (*p* = 0.851), whereas that of time was significant (*p* = 0.000; *p* = 0.008). Due to group–time interactions (*p* < 0.001), the pretest results for the control group were used as reference values for comparing how the groups differed in the interaction with time. In the experimental group, the mean score increased from 3.28 points in the pretest to 3.81 in the first posttest and then decreased to 3.39. In the control group, the corresponding scores decreased from 3.26 to 3.16 points and then further decreased to 2.97. According to GEE analysis, the first posttest score in the experimental group (*B* = 0.63, *p* < 0.001) and the follow-up test scores in the experimental group (*B* = 0.40, *p* < 0.001) were significantly higher than the pretest score in the control group. The run charts show that, by the end of the intervention, the mean scores for sense of hope in the experimental group had significantly increased—by 0.52 points (SD = 0.06). However, the effect of the horticultural activity intervention decreased with time; the corresponding score in the follow-up test was 0.41 points lower (SD *=* 0.05) than that in the first posttest. In the control group, mean scores for sense of hope gradually declined by 0.29 points from the pretest to the follow-up test. Table 6 shows the GEE results.

#### 3.5.3. Hand–Eye Coordination

In the follow-up test, the main effect of the group on hand–eye coordination was nonsignificant (*p* = 0.365), whereas the main effect of time was significant (*p* = 0.000). Because of group–time interactions (*p* < 0.001), the pretest results for the control group were used as reference values for comparing how the groups differed in the interaction with time. The mean cup stacking time in the experimental group decreased from 33.56 s in the pretest to 25.38 s in the first posttest. It then increased to 30.79 s in the follow-up test. The corresponding times in the control group increased from 36.45 s to 37.15 s and then to 41.72 s. GEE analysis revealed that cup stacking times for the experimental group in both the first posttest (*B* = −8.74, *p* < 0.001) and the follow-up test (*B* = −7.97, *p* < 0.001) were significantly shorter than that of the control group in the pretest. The curve chart for mean time in the two groups shows that, by the end of the intervention, the mean cup stacking time in the experimental group had decreased by 8.05 s (SD =1.16). However, the effect of the horticultural activity intervention gradually decreased with time; the corresponding time in the follow-up test was significantly longer (5.34 s longer; SD = 0.91) than that in the first posttest. The cup stacking time in the control gradually increased by 5.26 s from the pretest to the follow-up test. Table 6 shows the GEE results.

## 4. Discussion

Beyond being disease-free and having normal cognitive function, successful aging implies that older adults can generally continue to participate in society and engage in productive activities [63]. Because attitudes determine behavioral intention [64,65], attitudes toward aging affect how older adults contemplate the future and affect their problem-solving abilities in later stages of life. This study revealed that horticultural activities encouraged the development of positive attitudes toward aging, as reported previously in the literature [40,66]. Analysis of the three dimensions of attitudes toward aging revealed that, in addition to its cognitive and behavioral benefits, the horticultural program had emotional benefits, i.e., it induced a positive attitude about aging. In terms of the cognitive aspects of the intervention, the participants acquired knowledge in hydroponics and plant reproduction and learned to make cuttings of plants (e.g., Dracaena sanderiana and arrowhead vines). By using live plants as horticultural materials, the participants realized that new plants can be produced from only parts of the parent plant. This intervention, which has been widely used in relevant research (e.g., [67,68]), conveys to older adults that, although their physical condition is not as satisfactory as it was in their youth, they can still lead a happy life as long as they maintain a positive outlook on life. Regarding the emotional aspect, horticulture is a leisure activity favored by older people [69,70]. The researchers selected silvery wormwood (an herbaceous plant) and Yulan magnolia (an aromatic plant), both of which are common in rural Taiwan, to induce fond childhood memories in the participants. Upon touching or smelling the plants, the participants began to reminisce about their childhood. Plant scents reduce sympathetic nerve activity and increase parasympathetic nerve activity, which then cause feelings of comfort, relaxation, and happiness [35,71]. Physical and mental therapeutic effects of repeatedly working with flowering plants (e.g., eucalyptus, roses, and jasmine) in the program included positive attitudes toward aging. Regarding the behavioral aspects of the intervention, expression is one of the strongest motivators for older adults to learn [72,73]. Both the experimental and control groups scored highest on the item, “I want to set a good example for younger people”. This indicates that how they are perceived by the younger generation and how they interact with the younger generation are important to older Taiwanese adults. Horticultural activities not only offer older adults an opportunity to express themselves, but they also provide opportunities for social interaction [74]. The analysis of the first posttest scores confirmed the effectiveness of the interventions. Specifically, scores in the psychosocial loss dimension were lower in comparison with the pretest, whereas scores for psychological growth dimension were higher. This result is in line with the premise that horticultural therapy can enhance social participation among older adults [75,76]. Studies on the benefits of horticultural activities seldom discuss their effect on attitudes toward aging. In one study on Australian older adults [66], the trend of participant scores on the three dimensions of AAQ was consistent with that of the current study, in which psychological growth, physical change, and psychosocial loss were ranked first through third. The Australian study focused on understanding the relationship between the benefits of horticultural activities and attitudes toward aging, and it determined that the restoration benefits of horticultural activities can improve the psychological growth dimension of attitudes toward aging. In addition, the physical benefits of horticultural activities had a positive effect on the physical change dimension of attitudes toward aging. To be precise, older adults who obtained spiritual relaxation benefits from horticultural activities were more able to appreciate their aging process; older adults who maintained physical fitness through horticulture were more willing to accept their physical change in the aging process. The current study emphasized the persistence of the effect of horticultural therapy on attitudes toward aging. Relative to previous studies that provide directions for formulating teaching plans, the present study can be used as a reference for determining treatment duration.

Sense of hope is not only a motivation, but also a form of psychological energy characterized by expectations of positive outcomes of circumstances or events. Individuals with a sense of hope look forward to a promising future [77,78,79]. According to the literature, hope is fostered by interpersonal connectedness, attainable aims, a spiritual base, personal attributes, lightheartedness, uplifting memories, and affirmation of worth; in contrast, hope is undermined by abandonment and isolation, uncontrollable pain and discomfort, and devaluation of personhood [19]. Both before and after the present intervention, the item “I feel all alone” received the lowest score, suggesting that abandonment and isolation was the main factor in erosion of hope in participants. The HHI item with the highest score was item 9 (“I am able to give and receive caring and love”). Generally, institutional care, however high in quality or affection, cannot replace intimate family relationships. After the intervention, the participants scored significantly higher on items ‘’I have a positive outlook toward life” and “I have short- and/or long-range goals”. The higher rankings for these items after the intervention indicate that the intervention prompted the participants to set new goals. Studies have demonstrated that the benefits of horticultural therapy are exerted through interaction, action, and reaction. Specifically, interaction refers to connecting and engaging with others during horticultural activities, action refers to physical and mental horticultural efforts, and reaction refers to the impacts of plant growth on individuals [80]. Specifically, the wishes or expectations for their plants to thrive go on to influence other aspects of life [81]. That is, connecting with plant life is conducive to a sense of hope in older adults [82]. The program was designed to provide affirmation of worth in the participants by encouraging them to set easily achieved goals. For example, the teaching materials included plants that easily grow from seeds, e.g., bok choy and fern pine. The participants marveled at the germination process, and caring for the seedling gave them a sense of accomplishment, which strengthened their sense of hope. Additionally, professional guidance facilitated friendly interactions and interpersonal connectedness between the participants and the activity leader, which reduced the sense of rejection [83]. Based on the findings of other studies, these measures constitute a reasonable explanation for the significantly increased sense of hope observed in the participants after the intervention [67,84]. In studies of the benefits of horticultural activities, typically discussed psychological variables include depression, loneliness, and happiness; sense of hope is rarely discussed. In a South Korean study [67], the Dispositional Hope Scale [85] was used to evaluate sense of hope in rural women. This 4-point self-reporting scale was administered before and after 24 sessions of horticultural activities. The results indicated that the performance of the control group dropped from 3.40 (0.32) in the pretest to 3.39 (0.34) in the posttest, whereas the performance of the experimental group increased from 3.45 (0.34) to 3.61 (0.33). Although the measurement tool used in [67] differed from those in the present study, the data trends were consistent and confirmed that horticulture can indeed enhance sense of hope. A study of a Taiwanese community of older adults [86] used the HHI to evaluate the effects of a 12-week course of therapeutic horticultural activities. The score for a single item increased from 2.57 (0.63) to 2.96 (0.40) in the control group and from 2.34 (0.34) to 3.07 (0.19) in the experimental group. The study, which had a longer intervention period than the 8-week course of treatment in the current study, confirmed that horticultural activities effectively enhanced sense of hope in the experimental group. Notably, the posttest score in the control group did not decrease; rather, it slightly increased. However, the control group in the current study presented a downward trend in scores for hope. The reason may be that the older adults recruited for the current study were residents of care facilities, who often experience long-term separation from family members. Thus, these residents were vulnerable to feelings of abandonment and isolation, which may have decreased their sense of hope. As a result, sense of hope widely differed among the older adults of the community. Moreover, because the aforementioned study did not perform a second follow-up test, whether a longer course of treatment can achieve a more persistent effect remains uncertain. In brief, in addition to the duration of treatment, the background of the person being treated is a key factor in the benefits of horticulture.

As nervous system function and muscle strength deteriorate with age, hand–eye coordination is impaired [87,88]. Consistent with the present findings, multiple studies have reported that horticultural therapy effectively mitigates chronic musculoskeletal pain in older adults [67,87,88,89], protects the nervous system [57], enhances sensorimotor function [43], and improves hand–eye coordination [90,91]. A review of the relevant literature indicates that most studies have compared the relationship between horticultural activities and hand–eye coordination in individuals with and without disabilities. Courses designed for those without disabilities are generally delivered in 10 sessions [92] or 15 sessions [90]; measurement tools used for hand–eye coordination include digital grip dynamometer, Jamar hydraulic pinch gauge, and grooved pegboard. Multiple studies have noted significant improvements in the hand agility of participants who completed horticulture interventions. For example, the duration of grooved pegboard operation in experimental groups improved from 136.9 (69.3) to 133.5 (113.9) in [90] and from 80.6 (15.2) to 76.9 (13.6) in [38]. According to research data, grooved pegboard operation requires more time than cup stacking, possibly because the pegboard test requires greater use of fine motor skills. Moreover, the rate of improvement in grooved pegboard operation was lower than that in the cup stacking test. Possible explanations for these discrepancies include the longer testing time, the increased difficulty concentrating on the task in older adults, and the competitive nature of the cup stacking test, which may attract the interest of the participants. Notably, however, the accuracy of the cup stacking test for assessing grip and pinch strength is lower than that of the three aforementioned tools. One study reported that an 18-session horticultural therapy did not significantly improve hand dexterity in patients with disability (e.g., patients with stroke) [93]. Therefore, most researchers prefer to set a longer course of hand–eye coordination training for individuals with disability, e.g., 20 sessions [94] or 24 sessions [38] of training. Assessment of the effectiveness of a horticultural intervention should consider the characteristics of the group. Therefore, the selection of tools for measuring outcomes of horticultural therapy should consider not only the accuracy of the tool, but also psychological factors (e.g., achievement and motivation) in the participants.

According to a literature review, the effectiveness of methods for training hand–eye coordination have a strong correlation with age. For example, training methods for improving hand–eye coordination that involve repetitive movement possibly resulting in injuries (e.g., tennis, table tennis, and football) are most effective for younger adults [95,96,97,98]. However, in eastern countries, older adults prefer low-intensity activities over high-intensity activities [99]. Furthermore, studies indicate that activities combining physical and cognitive training are more effective at improving hand–eye coordination compared to those involving only physical or cognitive training [100,101,102]. Because emotion is an important factor in learning efficiency [102,103], overemphasis on physical training without the incorporation of leisure may cause older adults to lose interest and discontinue the activity. In contrast, learning efficiency increases in activities that learners find interesting or pleasant [104], which explains why training programs often integrate low-intensity activities (e.g., video games [105,106], tai chi [34,107], softball [108], mahjong [109], and flower arrangement [110]). Hand–eye coordination training programs of this nature (i.e., interactive and dynamic) are more effective for older adults than programs involving repetitive movement [111]. Therefore, the activities designed by the researchers in the current study incorporated multiple manual movements, including stringing flowers into a wreath, hill seeding, pasting, bouquet tying, flower arrangement, and rolling and cutting pieces of paper. That is, training in hand–eye coordination was integrated in entertaining horticultural activities. Therefore, the participants had high tolerance for the horticultural intervention activities because they provided a refreshing departure from the standard rehabilitation activities offered by medical institutions. The participants had significantly improved cup stacking speed after the intervention, which indicates that the intervention promoted hand–eye coordination. In both groups, the follow-up test scores for attitudes toward aging, sense of hope, and hand–eye coordination revealed significant interactions between group and time. In short, improvements in these outcomes were significantly greater in the experimental group than in the control group.

In summary, the participants scored lowest on the psychosocial loss and loneliness dimensions of the AAQ and HHI, respectively, which indicates that depression, feelings of emptiness, and feelings of isolation are pervasive among older adults in residential care facilities and that solutions are urgently needed. Notably, the significant positive correlation between psychosocial loss and age reported in an earlier study [66] is a reasonable explanation not only for the gradual reduction in the beneficial effects of the current intervention (i.e., attitudes toward aging and sense of hope) observed over time, but also for the higher scores in the follow-up test compared to the first posttest. Although the scores decreased, the therapeutic effects of the horticultural therapy in the experimental group continued for longer than 3 months, and follow-up test scores remained higher than pretest scores, which is in line with relevant studies [51,112]. This implies that horticultural activities have a direct long-term positive influence on health. Because of the limited land area and high population density of Taiwan, residential care facilities in cities rarely provide a sufficient area of green space for physical activity. The present program overcomes this obstacle because it can be implemented indoors and with only a small amount of green space. Additionally, the program is not subject to weather conditions. Therefore, it can be considered a practical and economical option for physical activity that can be implemented in care facilities of small or medium size in Taiwan.

A limitation of this study is that the participants were a convenience sample of older adults recruited from only three residential care facilities in a specific geographic area (Southern Taiwan). Although heterogeneity of baseline demographic characteristics between the experimental and control groups was not problematic in this study, future studies can increase the general ability of the findings, increasing the sample size as well as the frequency and duration of the intervention. Furthermore, to enhance data accuracy and to clarify the medical benefits of the intervention, investigation of other physiological indicators can be incorporated in the study design. Courses involving outdoor activities can also be incorporated in the program to assess physical changes beyond hand–eye coordination.

## 5. Conclusions

The 8-week horticultural program investigated in this study improved attitudes toward aging, hand–eye coordination, and sense of hope in the participants. Through professional guidance and experience sharing in groups, the program mitigated the sense of psychosocial loss in these elderly participants and induced psychological growth. The use of plant life as horticultural materials inspired a more positive, systematic, and hopeful approach to life in the participants. The wide range of manual movements required to complete the horticultural activities (including stringing, inserting, rolling, cutting, sticking, and binding motions) unconsciously induced development of hand–eye coordination skills in the participants. The effects of the intervention were still observable 3 months after its completion, which indicated the benefits of horticultural activities for enhancing sense of hope and hand–eye coordination in older adults and for inducing a positive attitude toward aging.

## Figures and Tables

**Table 1:** Content of the 8-week horticultural activity program.

Session	Topic	Activity Goals	Activity
1	Planting a Shade Tree	Improve hand–eye coordination. Develop positive attitudes toward aging and gain a sense of accomplishment from the completion of a project. Develop a sense of hope through the observation of plant growth.	Plant fern pine seeds in the planter through hill seeding, with the tip of the seeds pointed downward. Add some Maifan stones to secure the seeds and pour a sufficient amount of water over the soil.
2	Fragrant Wreath	Improve hand–eye coordination. Stimulate the olfactory nerve through the smelling of plant scents. Increase self-confidence and gain a sense of accomplishment from the completion of a project.	Cut the eucalyptus leaves into pieces. Penetrate the pedicel of a magnolia flower using a No. 20 iron wire. Then, string it together with the eucalyptus leaves. Repeat until all the flowers and leaves have been strung together. Bend the wire into a circle to form a door wreath.
3	Happy Farmer	Improve hand–eye coordination. Develop positive attitudes toward aging and gain a sense of accomplishment from the completion of a project. Develop a sense of hope through the observation of plant growth.	Poke holes in the bottom of a Styrofoam box with bamboo chopsticks and fill it with potting soil. Make a small seed bed and sow 3 to 5 bok choy seeds, spaced 5 cm apart. Cover the seeds with soil and water them.
4	Crystal World	Improve hand–eye coordination. Stimulate the optic nerve through the observation of colorful flowers and gel beads. Develop a sense of hope through the observation of plant growth.	Soak different colors of hydrogel crystal soil in a plastic bowl of water for 20 min to allow them to expand. Scoop the gel beads into a clear vase. Wash off the soil from the roots of an arrowhead vine. Place the plant into the vase and adjust the plant’s position.
5	Gift of Love	Improve hand–eye coordination. Stimulate the optic nerve through the observation of colorful flowers. Develop positive attitudes toward aging and gain a sense of accomplishment from the completion of a project.	Trim the stems of the dried flowers to different lengths (15–20 cm). Arrange the dried flowers to your preference into a bouquet. Cluster the flowers together with tape and envelop the bouquet in wrapping paper.
6	Sweet Talk	Improve hand–eye coordination. Stimulate the optic nerve through the observation of colorful flowers. Stimulate the olfactory nerve through the smelling of plant scents. Develop positive attitudes toward aging and gain a sense of accomplishment from the completion of a project.	Place the floral foam in a vase. Trim the stems or branches of the carnations, jasmine, baby’s breath, and other flowers to approximately 10 cm. Remove excess leaves from the stems before inserting them into the center and four corners of the foam so that the flowers form a circle. Fill in the gaps with leaves.
7	Longevity	Improve hand–eye coordination. Stimulate the optic nerve through the observation of colorful flowers. Develop positive attitudes toward aging and gain a sense of accomplishment from the completion of a project. Develop a sense of hope through the observation of plant growth	Peel away the outer leaves of the lucky bamboo (binomial name: dracaena sanderiana) and divide them into 12 cm sections, each of which must contain at least two nodes. Align the nodes on the upper end of the sections, with the buds facing outward. Tie the upper and lower ends of the sections with thin aluminum wires. Secure a red ribbon onto the upper end of the sections before placing them in a glass jar for regular watering.
8	The smell reaches far	Improve hand–eye coordination. Stimulate the olfactory nerve through the smelling of plant scents. Develop positive attitudes toward aging and gain a sense of accomplishment from the completion of a project.	Cut a piece of rice paper into a rectangle shape and roll it into a cylinder using a permanent marker. Tightly twist one end of the cylinder before withdrawing the marker. Tightly pack the cylinder with dried wormwood using a bamboo stick. Then, tightly twist the other end of the cylinder to form a coil.

**Table 2:** Results of the chi-squared test of baseline demographic characteristics in the experimental and control groups.

Demographic Characteristics Variables	Control Group	Experimental Group	*χ^2^*	*p*
	*n (%)*	*n (%)*		
Sex				
Male	19 (44.2)	20 (44.4)	0.00	0.98
Female	24 (55.8)	25 (55.6)		
Age (years)				
65 and below	2 (4.7)	2 (4.4)	5.82	0.21
66–70	4 (9.3)	10 (22.2)		
71–75	15 (34.9)	12 (26.7)		
76–80	17 (39.5)	11 (24.4)		
Above 81	5 (11.6)	10 (22.2)		
Education level				
Elementary school and No formal education	30 (69.8)	32 (71.1)	0.17	0.92
Junior high school	7 (16.3)	6 (13.3)		
High school	6 (14.0)	7 (15.6)		
Religion				
None	12 (27.9)	8 (17.8)	1.95	0.74
Buddhism	14 (32.6)	20 (44.4)		
Taoism	10 (23.3)	11 (24.4)		
Christianity	6 (14.0)	5 (11.1)		
Catholic	1 (2.3)	1 (2.2)		
Marital status				
Single	15 (34.9)	10 (22.2)	1.74	0.42
Married	9 (20.9)	11 (24.4)		
Widowed	19 (44.2)	24 (53.3)		
Children				
Yes	30 (69.8)	33 (73.3)	0.14	0.71
No	13 (30.2)	12 (26.7)		
Disease history				
None	5 (11.6)	3 (6.8)	4.59	0.33
Heart Disease	9 (20.9)	11 (25.0)		
Hypertension	10 (23.3)	18 (40.9)		
Diabetes	12 (27.9)	8 (18.2)		
Osteoporosis	7 (16.3)	4 (9.1)		
Psychosocial variables	Mean (SD)	Mean (SD)	*t*-value	*p*
Attitudes toward aging	3.75 (0.27)	3.81 (0.24)	−1.254	0.21
Sense of hope	3.26 (0.54)	3.28 (0.49)	−0.182	0.86
Hand–eye coordination	36.45 (15.61)	33.56 (15.51)	0.871	0.39

**Table 3:** Scores for individual items of the Attitudes to Ageing Questionnaire, the hope Herth index, and the hand–eye coordination test.

Variables	T0	Ranking of Grades	T1	Ranking of Grades	T2	Ranking of Grades
	Mean (SD)		Mean (SD)		Mean (SD)	
Aging attitudes						
Control group	3.75 (0.27)	2	3.70 (0.28)	2	3.48 (0.34)	2
Experimental group	3.81 (0.24)	1	4.74 (0.27)	1	4.31 (0.25)	1
Aging attitudes—Psychosocial loss (These items have been scored in reverse)						
Control group	2.75 (0.61)	1	2.72 (0.76)	2	2.49 (0.55)	2
Experimental group	2.35 (0.54)	2	4.56 (0.54)	1	3.52 (0.71)	1
1. Old age is a time of loneliness.						
Control group	2.65 (0.75)	20	2.69 (0.89)	22	2.42 (0.63)	22
Experimental group	2.24 (0.71)	20	4.47 (0.73)	22	3.04 (0.98)	24
2. Old age is a depressing time of life.						
Control group	2.60 (0.76)	22	2.84 (0.97)	17	2.53 (0.80)	19
Experimental group	2.24 (0.68)	20	4.58 (0.58)	18	3.09 (1.02)	22
3. I find it more difficult to talk about my feelings as I get older.						
Control group	2.58 (0.73)	24	2.70 (0.89)	21	2.42 (0.59)	22
Experimental group	2.16 (0.60)	23	4.62 (0.53)	17	3.08 (1.01)	23
4. I see old age mainly as a time of loss.						
Control group	2.63 (0.79)	21	2.81 (0.85)	20	2.56 (0.59)	18
Experimental group	2.16 (0.60)	23	4.56 (0.62)	20	3.51 (0.66)	21
5. I am losing my physical independence as I get older.						
Control group	2.60 (0.79)	22	2.65 (0.95)	23	2.47 (0.80)	21
Experimental group	2.18 (0.58)	22	4.53 (0.66)	21	3.58 (0.72)	20
6. As I get older, I find it more difficult to make new friends.						
Control group	2.86(0.86)	19	2.72 (0.96)	19	2.49 (0.86)	20
Experimental group	2.56(0.76)	18	4.38 (0.89)	23	3.78 (0.90)	19
7. I don’t feel involved in society now that I am older.						
Control group	2.98 (0.83)	18	2.53 (1.03)	24	2.42 (0.79)	22
Experimental group	2.53 (0.63)	19	4.69 (0.51)	15	4.00 (0.88)	18
8. I feel excluded from things because of my age.						
Control group	3.07 (0.88)	17	2.79 (0.89)	18	2.60 (0.69)	17
Experimental group	2.71 (0.69)	17	4.67 (0.52)	16	4.09 (0.82)	17
Aging attitudes—Physical change						
Control group	4.08 (0.54)	2	4.10 (0.52)	2	3.90 (0.56)	2
Experimental group	4.33 (0.44)	1	4.75 (0.34)	1	4.71 (0.30)	1
9. It is important to take exercise at any age.						
Control group	4.60 (0.76)	2	4.60 (0.79)	3	4.28 (0.73)	4
Experimental group	4.73 (0.54)	7	4.36 (1.03)	24	4.73 (0.65)	5
10. Growing older has been easier than I thought.						
Control group	3.88 (0.54)	15	3.93 (0.86)	15	3.86 (0.86)	8
Experimental group	4.00 (0.37)	15	4.58 (0.62)	18	4.82 (0.39)	4
11. I don’t feel old.						
Control group	3.93 (0.80)	13	3.98 (0.86)	13	3.79 (0.86)	11
Experimental group	3.91 (1.00)	16	4.78 (0.42)	13	4.87 (0.34)	2
12. My identity is not defined by my age.						
Control group	4.09 (0.65)	11	4.05 (0.62)	9	3.98 (0.60)	6
Experimental group	4.42 (0.50)	11	4.82 (0.39)	12	4.56 (0.50)	15
3. I have more energy now than I expected for my age.						
Control group	4.02 (0.67)	12	4.05 (0.58)	9	3.91 (0.53)	7
Experimental group	4.38 (0.53)	13	4.84 (0.37)	11	4.71 (0.46)	6
14. Problems with my physical health do not hold me back from doing what I want to.						
Control group	3.83 (0.84)	16	4.07 (0.63)	8	3.83 (0.57)	10
Experimental group	4.02 (0.89)	14	4.87 (0.34)	9	4.69 (0.47)	7
15. My health is better than I expected for my age.						
Control group	3.93 (0.88)	13	4.00 (0.69)	12	3.72 (0.59)	14
Experimental group	4.40 (0.50)	12	4.87 (0.34)	9	4.64 (0.48)	11
16. I keep myself as fit and active as possible by exercising.						
Control group	4.30 (0.74)	8	4.16 (0.75)	6	3.79 (0.74)	11
Experimental group	4.80 (0.40)	3	4.89 (0.32)	5	4.64 (0.48)	11
Aging attitudes—Psychological growth						
Control group	4.42 (0.58)	2	4.27 (0.48)	2	4.06 (0.57)	2
Experimental group	4.76 (0.36)	1	4.90 (0.16)	1	4.70 (0.29)	1
17. As people get older, they are better able to cope with life.						
Control group	4.41 (0.66)	5	4.30 (0.56)	5	3.86 (0.60)	8
Experimental group	4.80 (0.40)	3	4.71 (0.46)	14	4.53 (0.50)	16
18. It is a privilege to grow old.						
Control group	4.26 (0.76)	9	3.88 (0.70)	16	3.60 (0.58)	16
Experimental group	4.76 (0.43)	6	4.96 (0.21)	2	4.60 (0.50)	13
19. Wisdom comes with age.						
Control group	4.14 (0.97)	10	4.02 (0.80)	11	3.65 (0.69)	15
Experimental group	4.69 (0.60)	8	4.89 (0.49)	5	4.67 (0.48)	9
20. There are many pleasant things about growing older.						
Control group	4.33 (0.68)	6	3.98 (0.74)	13	3.77 (0.75)	13
Experimental group	4.60 (0.58)	10	4.96 (0.21)	2	4.60 (0.50)	13
21. I am more accepting of myself as I have grown older.						
Control group	4.33 (0.71)	6	4.09 (0.75)	7	4.07 (0.86)	5
Experimental group	4.62 (0.58)	9	4.96 (0.21)	2	4.69 (0.47)	7
22. It is very important to pass on the benefits of my experiences to younger people.						
Control group	4.58 (0.59)	3	4.63 (0.54)	2	4.47 (0.63)	2
Experimental group	4.93 (0.25)	1	4.89 (0.38)	5	*4.87 (0.34)*	2
23. I believe my life has made a difference.						
Control group	4.56 (0.55)	4	4.46 (0.55)	4	4.33 (0.78)	3
Experimental group	4.78 (0.42)	5	4.89 (0.38)	5	4.67 (0.48)	9
24. I want to give a good example to younger people.						
Control group	4.74 (0.44)	1	4.81 (0.45)	1	4.70 (0.51)	1
Experimental group	4.93 (0.25)	1	4.98 (0.15)	1	4.98 (0.15)	1
Sense of hope						
Control group	3.26 (0.54)	2	3.16 (0.48)	2	2.97 (0.41)	2
Experimental group	3.28 (0.49)	1	3.81 (0.26)	1	3.39 (0.40)	1
Sense of hope—inner sense of temporality and future						
Control group	3.15 (0.63)	1	2.97 (0.48)	2	2.83 (0.55)	2
Experimental group	3.08 (0.55)	2	3.72 (0.35)	1	3.32 (0.46)	1
1. I have a positive outlook toward life.						
Control group	3.28 (0.88)	7	3.07 (0.70)	9	2.88 (0.66)	6
Experimental group	3.24 (0.83)	12	3.89 (0.38)	6	3.62 (0.53)	3
2. I have short- and/or long-range goals.						
Control group	3.19 (0.85)	9	3.05 (0.69)	10	2.86 (0.68)	7
Experimental group	3.25 (0.83)	11	3.89 (0.38)	6	3.60 (0.58)	5
6. I feel scared about my future. (The item has been scored in reverse)						
Control group	2.86 (0.86)	11	2.53 (0.88)	11	2.72 (0.93)	11
Experimental group	2.36 (0.88)	8	3.22 (0.82)	11	2.56 (0.76)	11
11. I believe that each day has potential.						
Control group	3.28 (0.80)	7	3.21 (0.83)	8	2.83 (0.53)	9
Experimental group	3.47 (0.76)	5	3.89 (0.32)	6	3.51 (0.55)	7
Sense of hope—inner positive readiness and expectancy						
Control group	3.32 (0.63)	2	3.37 (0.65)	2	3.01 (0.41)	2
Experimental group	3.44 (0.61)	1	3.93 (0.25)	1	3.53 (0.43)	1
4. I can see possibilities in the midst of difficulties.						
Control group	3.37 (0.90)	5	3.33 (0.80)	5	3.02 (0.77)	5
Experimental group	3.44 (0.69)	6	3.93 (0.25)	4	3.62 (0.53)	3
7. I can recall happy/joyful times.						
Control group	3.44 (0.73)	3	3.49 (0.59)	1	3.40 (0.49)	2
Experimental group	3.62 (0.53)	3	4.00 (0.00)	1	3.78 (0.47)	2
10. I have a sense of direction.						
Control group	3.07 (0.63)	10	3.35 (0.72)	4	2.86 (0.52)	7
Experimental group	3.29 (0.76)	10	3.89 (0.38)	6	3.27 (0.54)	10
12. I feel my life has value and worth.						
Control group	3.40 (0.79)	4	3.30 (0.80)	6	2.77 (0.57)	10
Experimental group	3.42 (0.78)	7	3.89 (0.38)	6	3.47 (0.59)	9
Sense of hope—inter-connectedness with self and others						
Control group	3.31 (0.50)	2	3.13 (0.44)	2	3.08 (0.47)	2
Experimental group	3.32 (0.43)	1	3.77 (0.26)	1	3.33 (0.37)	1
3. I feel all alone. (The item has been scored in reverse)						
Control group	2.79 (0.86)	12	2.37 (0.72)	12	2.49 (0.77)	12
Experimental group	2.33 (0.90)	9	3.20 (0.79)	12	2.33 (0.48)	12
5. I have a faith that gives me comfort.						
Control group	3.48 (0.80)	2	3.23 (0.90)	7	3.19 (0.73)	3
Experimental group	3.71 (0.59)	2	3.96 (0.21)	3	3.60 (0.54)	5
8. I have deep inner strength.						
Control group	3.37 (0.72)	5	3.44 (0.67)	3	3.14 (0.60)	4
Experimental group	3.51 (0.66)	4	3.93 (0.25)	4	3.51 (0.59)	7
9. I am able to give and receive caring and love.						
Control group	3.60 (0.58)	1	3.49 (0.63)	1	3.51 (0.63)	1
Experimental group	3.73 (0.54)	1	3.98 (0.15)	2	3.87 (0.40)	1
Hand–eye coordination						
Control group	36.45 (15.61)	2	37.15 (15.47)	2	41.72 (14.97)	2
Experimental group	33.56 (15.51)	1	25.38 (12.38)	1	30.79 (11.50)	1

**Table 4:** Effect size (Cohen’s *d*) of the intervention on attitudes toward aging, sense of hope, and hand–eye coordination.

Variable	First Posttest	Cohen’s *d*	Follow-Up Test	Cohen’s *d*
Attitudes toward Aging				
Control group	3.70 ± 0.28	3.78	3.48 ± 0.34	2.78
Experimental group	4.74 ± 0.27		4.31 ± 0.25	
Sense of hope				
Control group	3.16 ± 0.48	1.68	2.97 ± 0.41	1.04
Experimental group	3.81 ± 0.26		3.39 ± 0.40	
Hand–eye coordination				
Control group	37.15 ± 15.47	−0.84	41.72 ± 14.97	−0.82
Experimental group	25.38 ± 12.38		30.79 ± 11.50	

Data are expressed as the mean ± standard deviation. The effect size is reported by Cohen’s *d*.

**Table 5:** Pearson product–moment correlation analysis of attitudes toward aging, sense of hope, and hand–eye coordination.

Pretest Correlation Coefficient (r Value) of the Control Group									
Variable	1	2	3	4	5	6	7	8	9
1. Attitudes toward aging	1								
2. Psychosocial loss	0.068	1							
3. Physical change	0.771 **	−0.382 *	1						
4. Psychological growth	0.622 **	−0.609 **	0.564 **	1					
5. Sense of hope	−0.043	0.086	0.043	−0.192	1				
6. Inner sense of temporality and future	0.24	0.125	0.087	−0.179	0.951 **	1			
7. Inner positive readiness and expectancy	−0.034	0.059	0.068	−0.173	0.922 **	0.836 **	1		
8. Interconnectedness with self and others	−0.126	0.047	−0.054	−0.177	0.864**	0.753 **	0.662 **	1	
9. Hand–eye coordination	−0.307 *	−0.051	−0.193	−0.200	−0.002	−0.009	−0.010	0.015	1
**First Posttest Correlation Coefficient (r Value) of the Control Group**									
1. Attitudes toward aging	1								
2. Psychosocial loss	0.319 *	1							
3. Physical change	0.615 **	−0.467 **	1						
4. Psychological growth	0.549 **	−0.525 **	0.711**	1					
5. Sense of hope	−0.082	0.035	−0.106	−0.081	1				
6. Inner sense of temporality and future	−0.036	−0.023	−0.036	−0.013	0.840 **	1			
7. Inner positive readiness and expectancy	−0.087	0.068	−0.128	−0.118	0.951 **	0.668 **	1		
8. Interconnectedness with self and others	−0.099	0.039	−0.117	−0.105	0.930 **	0.652 **	0.890 **	1	
9. Hand–eye coordination	−0.310 *	−0.219	−0.096	−0.084	0.084	−0.044	0.153	0.097	1
**Follow-Up Test Correlation Coefficient (r Value) of the Control Group**									
1. Attitudes toward aging	1								
2. Psychosocial loss	0.139	1							
3. Physical change	0.843 **	−0.330 *	1						
4. Psychological growth	0.815 **	−0.385 *	0.831 **	1					
5. Sense of hope	0.115	−0.053	0.139	0.118	1				
6. Inner sense of temporality and future	0.154	−0.059	0.145	0.187	0.865 **	1			
7. Inner positive readiness and expectancy	0.097	−0.080	0.177	0.074	0.8311 **	0.536 **	1		
8. Interconnectedness with self and others	0.038	0.001	0.042	0.025	0.891 **	0.636 **	0.685 **	1	
9. Hand–eye coordination	−0.367 *	−0.236	−0.162	−0.268	0.013	−0.064	0.186	−0.053	1
**Pretest Correlation Coefficient (r Value) of the Experimental Group**									
1. Attitudes toward aging	1								
2. Psychosocial loss	0.390 **	1							
3. Physical change	0.744 **	−0.196	1						
4. Psychological growth	0.464 **	−0.487**	0.520 **	1					
5. Sense of hope	0.144	0.109	−0.016	0.140	1				
6. Inner sense of temporality and future	0.157	0.105	0.011	0.187	0.950 **	1			
7. Inner positive readiness and expectancy	0.161	0.140	0.026	0.076	0.930 **	0.826 **	1		
8. Interconnectedness with self and others	0.063	0.040	0.042	0.193	0.882 **	0.796 **	0.701 **	1	
9. Hand–eye coordination	−0.117	−0.076	−0.066	−0.034	0.040	−0.002	−0.011	0.153	1
**First Posttest Correlation Coefficient (r Value) of the Experimental Group**									
1. Attitudes toward aging	1								
2. Psychosocial loss	0.868 **	1							
3. Physical change	0.719 **	0.319 *	1						
4. Psychological growth	0.596 **	0.321 *	0.442 **	1					
5. Sense of hope	0.054	0.075	0.019	−0.021	1				
6. Inner sense of temporality and future	0.111	0.142	0.036	0.001	0.978 **	1			
7. Inner positive readiness and expectancy	−0.152	−0.178	−0.017	−0.129	0.842 **	0.737 **	1		
8. Interconnectedness with self and others	0.160	0.207	0.024	0.057	0.938 **	0.940 **	0.628 **	1	
9. Hand–eye coordination	0.066	0.045	0.197	−0.237	−0.106	−0.084	−0.047	−0.163	1
**Follow-Up Test Correlation Coefficient (r Value) of the Experimental Group**									
1. Attitudes toward aging	1								
2. Psychosocial loss	0.793 **	1							
3. Physical change	0.164	−0.368 *	1						
4. Psychological growth	0.475 **	−0.031	0.307 *	1					
5. Sense of hope	0.229	0.207	−0.052	0.139	1				
6. Inner sense of temporality and future	0.229	0.195	−0.039	0.156	0.946 **	1			
7. Inner positive readiness and expectancy	0.244	0.228	−0.055	0.128	0.925 **	0.783 **	1		
8. Interconnectedness with self and others	0.167	0.156	−0.053	0.103	0.955 **	0.880 **	0.832 **	1	
9. Hand–eye coordination	−0.106	−0.142	0.124	−0.052	0.004	0.066	0.002	−0.070	1

* *p* < 0.05 and ** *p* < 0.01. Correlation coefficient <0.3 denotes low correlation, 0.3–0.7 denotes moderate correlation, and >0.7 denotes high correlation.

**Table 6:** Generalized estimating equation analysis results of attitudes toward aging in the experimental and control groups before and after the experiment.

	Aging Attitudes				Sense of Hope				Hand–Eye Coordination			
Variables	Mean (SD)	*B*	*SE*	*p*–Value	Mean (SD)	*B*	*SE*	*p*–Value	Mean (SD)	*B*	*SE*	*p*–Value
Intercept		3.75	0.04	<0.000 ***		3.26	0.08	<0.000 ***		36.46	2.35	<0.000 ***
Group (EG vs. CG)		0.07	0.05	0.208		0.02	0.11	0.851		−2.96	3.27	0.365
Time overall (T2 vs. T0)		−0.27	0.03	<0.000 ***		−0.29	0.05	<0.000 ***		5.26	1.27	<0.000 ***
Time overall (T1 vs. T0)		−0.05	0.02	0.047 *		−0.11	0.04	0.008 **		0.69	0.96	0.470
EG at T2	4.31 (0.04)				3.39 (0.06)				30.79 (1.69)			
EG at T1	4.74 (0.04)				3.81 (0.04)				25.45 (1.82)			
EG at T0	3.81 (0.03)				3.28 (0.07)				33.50 (2.27)			
CG at T2	3.48 (0.05)				2.97 (0.06)				41.72 (2.26)			
CG at T1	3.70 (0.04)				3.16 (0.07)				37.15 (2.33)			
CG at T0	3.75 (0.04)				3.26 (0.08)				36.45 (2.35)			
EG at T2 vs. EG at T1	−0.43 (0.05)			<0.001 ***	−0.41 (0.05)			<0.001 ***	5.34 (0.91)			<0.001 ***
EG at T1 vs. EG at T0	0.92 (0.04)			<0.001 ***	0.52 (0.06)			<0.001 ***	−8.05 (1.16)			<0.001 ***
CG at T2 vs. CG at T1	−0.22 (0.03)			<0.001 ***	−0.19 (0.03)			<0.001 ***	4.57 (1.02)			<0.001 ***
CG at T1 vs. CG at T0	−0.05 (0.02)			0.047 *	−0.10 (0.04)			0.209	0.69 (0.96)			0.470
Group*Time overall												
EG*(T2 vs. T0) vs. CG*(T2 vs. T0) a		0.77	0.06	<0.000 ***		0.40	0.08	<0.000 ***		−7.97	1.72	<0.000 ***
EG*(T1 vs. T0) vs. CG*(T1 vs. T0) b		0.97	0.05	<0.000 ***		0.63	0.07	<0.000 ***		−8.74	1.50	<0.000 ***

GEE, generalized estimating equation. T0, baseline; T1, at the end of the intervention (first posttest); T2, at the end of the intervention (follow-up test). EG, experimental group; CG, control group. a = [Experimental group (T1–T0)] − [Comparison group (T1–T0)]; b = [Experimental group (T2–T0)] − [Comparison group (T2–T0)]. * *p* < 0.05; ** *p* < 0.01; *** *p* < 0.001.

## Data Availability

The data presented in this study are available on request from the corresponding author. The data are not publicly available due to ethical restrictions.
